# Mask-R$^{2}$CNN: a distance-field regression version of Mask-RCNN for fetal-head delineation in ultrasound images

**DOI:** 10.1007/s11548-021-02430-0

**Published:** 2021-06-22

**Authors:** Sara Moccia, Maria Chiara Fiorentino, Emanuele Frontoni

**Affiliations:** 1 The BioRobotics Institute, Scuola Superiore Sant’Anna, Pisa, Italy; 2 Department of Excellence in Robotics and AI, Scuola Superiore Sant’Anna, Pisa, Italy; 3 Department of Information Engineering, Università Politecnica delle Marche, Ancona, Italy

**Keywords:** Deep learning, Distance fields, Fetal Ultrasound, Head-circumference delineation

## Abstract

**Background and objectives:**

Fetal head-circumference (HC) measurement from ultrasound (US) images provides useful hints for assessing fetal growth. Such measurement is performed manually during the actual clinical practice, posing issues relevant to intra- and inter-clinician variability. This work presents a fully automatic, deep-learning-based approach to HC delineation, which we named Mask-R$^{2}$CNN. It advances our previous work in the field and performs HC distance-field regression in an end-to-end fashion, without requiring a priori HC localization nor any postprocessing for outlier removal.

**Methods:**

Mask-R$^{2}$CNN follows the Mask-RCNN architecture, with a backbone inspired by feature-pyramid networks, a region-proposal network and the ROI align. The Mask-RCNN segmentation head is here modified to regress the HC distance field.

**Results:**

Mask-R$^{2}$CNN was tested on the *HC18 Challenge* dataset, which consists of 999 training and 335 testing images. With a comprehensive ablation study, we showed that Mask-R$^{2}$CNN achieved a mean absolute difference of 1.95 mm (standard deviation $=\pm 1.92$ mm), outperforming other approaches in the literature.

**Conclusions:**

With this work, we proposed an end-to-end model for HC distance-field regression. With our experimental results, we showed that Mask-R$^{2}$CNN may be an effective support for clinicians for assessing fetal growth.

## Introduction

Measuring fetal-head circumference (HC) is a common task in the clinical practice for assessing fetal growth. Ultrasound (US) imaging is the elected imaging modality for such assessment due to its accessibility and safety. Nowadays, HC measurement is performed manually by gynecologists, which delineate the fetal skull or select skull landmarks on the US image. Such procedure is time consuming and may be prone to intra- and inter-clinician variability [1]. To attenuate these issues, the medical-image analysis community has worked in the last decades to develop algorithms for automatic HC measurement from US images. This automatic measurement relies on HC delineation, which is a challenging task. US images present a low signal-to-noise ratio (with the presence of shadows and specking in the image), possibly resulting in missing edges [1]. For fetuses of the same gestational trimester, fetal HC varies among fetuses in terms of skull thicknesses and head size, with different contrast levels from background tissues. Such variability is further increased among fetuses of different gestational trimesters. As an additional challenge for automatic delineation algorithms, HC only covers a small portion of the US image.

To tackle the challenges of structure delineation in different fields, recent work [2, 3] in the literature has modeled the delineation problem as a heatmap-regression tasks, where a convolutional neural network (CNN) is used to regress a distance field from the contour to be delineated. Following such paradigm, in our previous work [4] we presented a two-step HC distance-field regression approach to fetal head delineation, which involves fetal-head localization with the Yolo network followed by an encoder-decoder CNN for HC distance-field regression. In fact, we showed that performing fetal head localization prior regressing the distance fields significantly improves the delineation performance.

**Fig. 1:**
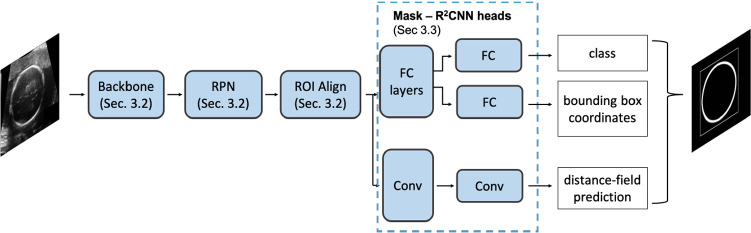
Mask-R$^2$CNN schematic architecture. Mask-R$^2$CNN predicts the head-circumference distance field. The relative bounding box is shown for visualization purposes. *RPN* region proposal network, *FC* fully connected layers, *Conv* convolutional layers

In this paper we move forward with respect to [4] and hypothesize that Mask-RCNN [5], which was originally developed for semantic-segmentation tasks, can be used to provide accurate regression of HC distance fields with an end-to-end approach. The main contribution of this work is a unified approach, called Mask-R$^2$CNN (Fig. 1), for fetal-HC delineation in US images. Our approach modifies the original Mask-RCNN by replacing the Mask-RCNN segmentation head with a new head for distance-field regression. Considering that our network regresses the distance field, we call it Mask-R$^{2}$CNN because one “R” refers to the “region” proposal approach followed by the standard Mask-RCNN and the other “R” refers to the distance-field regression task. The main innovation introduced here is that our approach regresses distance fields, instead of predicting a segmentation mask as in the original implementation of Mask-RCNN. Opposite to [4], the region proposal network (RPN) inherited from Mask-R$^2$CNN avoids the need of a priori HC localization. Furthermore, with our experiments that are carried out using the publicly available dataset released during the *HC18 Grand Challenge*,^1^ we show that the Mask-R$^2$CNN does not produce spurious prediction. This avoids the need for a posteriori outlier removal, making our model independent from the definition of post-processing parameters. Mask-R$^2$CNN is therefore easily generalizable to other datasets without any modification. The end-to-end approach further allows us simplifying and speeding up the training process, which is an important aspect to be considered with a view to collect more US images. At the same time, having a single stage, end-to-end architecture will allow to easily embed the algorithm in US machines.

### State of the art

Researchers in the medical-image community have been working in the last couple of decades for providing algorithms for automatic HC delineation. In 2018, the *HC18 Grand Challenge* was organized, with the release of a dataset of 1334 US images. Such dataset size, coupled with the growing availability of computational power, unlocked the potential of deep learning in the field. As a preliminary step for HC delineation, a number of researchers uses CNNs for segmenting the fetal head. The work in [6] proposes a CNN inspired by LinkNet to segment the fetal head and obtain, thought adding fully connected neurons, the HC main axes, center and angle. However, the problem of directly regressing measurements may be challenging, posing issues relevant to overfitting. The work in [7] uses UNet-like CNNs for head segmentation, showing interesting preliminary results on small custom datasets. In [8], Mask R-CNN is used for jointly localizing and segmenting the fetal head. The *HC18 Grand Challenge* dataset is used in combination with a custom dataset of more than 2000 images in [9] to train a probabilistic UNet. As a result, multiple HC segmentation hypotheses are provided to the clinicians, which can choose the best one. Segmentation CNNs with attention mechanism are investigated in [10, 11], showing interesting preliminary results.

**Fig. 2:**
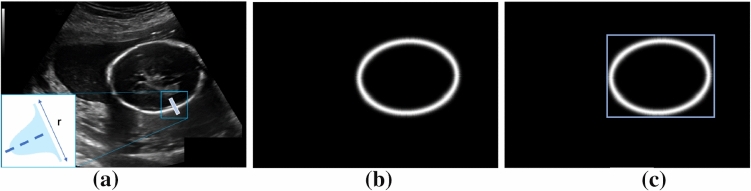
**a** Gaussian profile for building the distance-field regression ground truth, **b** distance-field regression ground truth, **c** visual representation of the bounding box ground-truth superimposed on the distance-field regression ground truth (the bounding box is thickened for visualization purposes)

Most of these approaches addresses the problem of HC delineation through fetal-head segmentation. In [4], the problem is addressed from a different perspective, by training a CNN to regress a distance field from the HC. However, the approach relies on a two-step approach for fetal head localization and distance-field regression, which is achieved by modifying UNet to accomplish a regression task. The regression network is then followed by a parameter-sensitive post-processing to discard outliers. In this work, we instead provide a unified framework for HC distance-field regression, which does not require any a priori HC localization nor time-consuming or parameter-sensitive post-processing.

## Method

The proposed strategy to train Mask-R$^2$CNN relies on distance fields. As introduced in section “Introduction”, the rationale behind using distance fields is to smooth the HC line as to facilitate the network task as opposed to directly regressing the HC line. To build the distance-field ground truth, we start from the HC annotation provided by the *HC18 Grand Challenge*, which consists of 2-pixels wide ellipses. We skeletonize the ellipses prior building our distance-field ground truth. Inspired by [4], we consider a region (Fig. 2) consisting of all pixels that lie in the rectangular region with thickness *r* pixels, centrally aligned with each of the pixel of the skeletonized HC, and perpendicular to the tangent of the HC. Each region is built to have a Gaussian intensity profile with standard deviation *r*/2. The bounding-box ground truth is then delineated to completely contain the distance-field ground truth.

The backbone, RPN and ROI align of Mask-R$^2$CNN follow the standard implementation of Mask-RCNN [5]. The backbone of Mask-R$^2$CNN is a feature pyramid network (FPN) that relies on ResNet-101. We chose this configuration as it achieved the best performance in [5]. The input US image is hence processed via a sequence of convolution and pooling. The convolutional and identity blocks follow the original implementation of ResNet [12]. The resulting feature maps (C1, C2, C3, C4, C5) are further processed by a top-down pathway with lateral connections. Convolutions in the pathway are performed with 256 $1\times 1$ filters. Up-sampling is performed with $2\times 2$ kernels and max pooling with pool size $1\times 1$ and strides 2. The output feature maps (P2, P3, P4, P5, P6) are processed by the RPN to generate candidates ROIs. The RPN anchors span 5 scales and 3 aspect ratios, to account for different HC size and ellipticity. Prior entering the Mask-R$^2$CNN heads, P2, P3, P4, P5, P6 are processed by the ROI align, which resizes the candidate ROIs to guarantee that all ROIs are squared and have the same (small) spatial size $d\times d$.

**Fig. 3:**
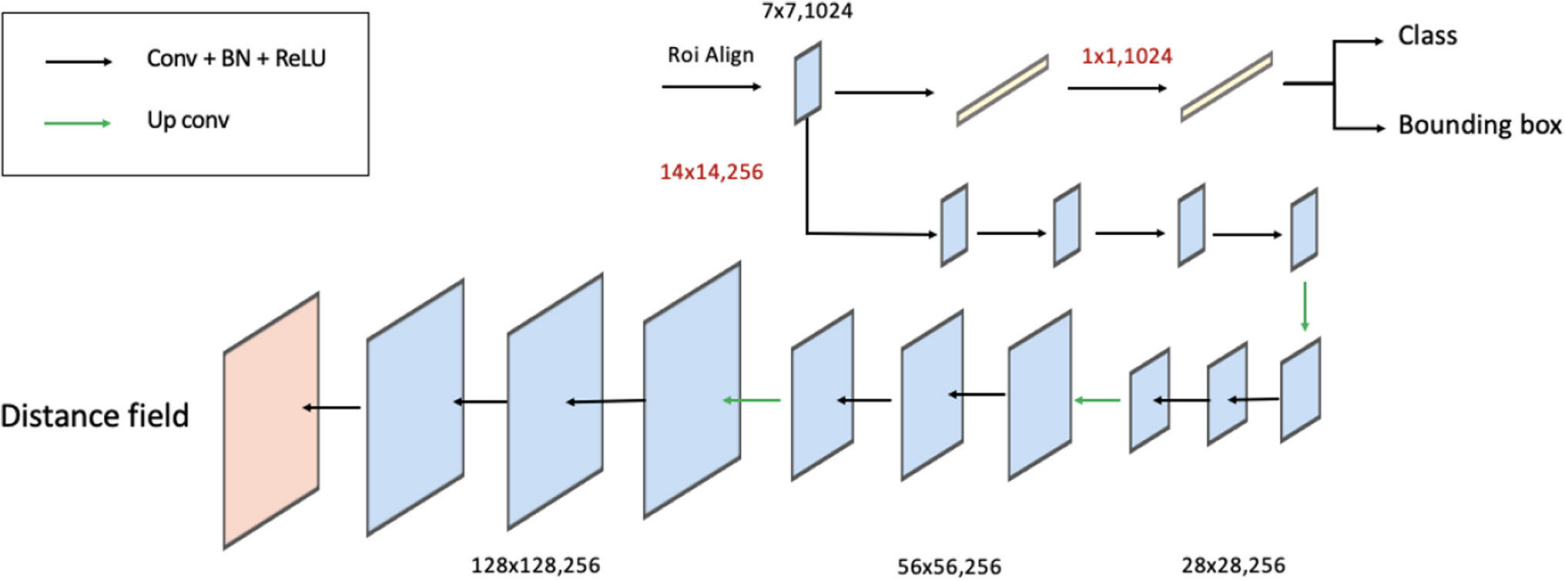
Mask-R$^2$CNN heads. *Conv* convolution, *Up conv* up-sampling $+$ convolution. *Red* convolution specification (kernel dimension, number of filters), *black* feature-map size

### Mask-R$^2$CNN heads

Mask-R$^2$CNN has three heads: the classification, bounding-box and distance-field regression heads, all fed with the ROI candidates from the ROI align (Fig. 3). In the classification and bounding-box heads, the ROI-aligned candidates are processed by two fully-connected layers with 1024 neurons. The classification head has a third fully-connected layer with 2 neurons (for fetal head and background), followed by softmax. The bounding-box head has a fully-connected layer with 4 neurons, linearly activated, which predict the anchor correction factors for the fetal-head class.

The architecture of the distance-field regression head is summarized in Table 1. The first four convolutions follow the implementation of the mask head of Mask RCNN. We replace the upsampling path of Mask RCNN, which originally consisted of a single transposed convolution with 256 $2\times 2$ filters with stride 2, with a sequence of up-convolutions. The upsampling path takes inspiration from the decoder path of Unet, which we used in our previous work for HC delineation [4]. Using the decoder path of Unet instead of a single transposed convolution allows us to restore the resolution of the distance field, and achieve an accurate prediction. Up-convolution is achieved with $2\times 2$ upsampling followed by convolution with 256 $2\times 2$ filters. We set the number of up-convolutions to 3, as a trade-off between regression performance and computational effort. Each up-convolution is followed by 2 $3\times 3$ convolutions with 256 filters. All convolutions are followed by batch normalization and activation with the rectified linear unit (ReLU). All convolutions in the Mask-R$^2$CNN heads are performed in parallel for all ROIs using time-distributed convolution.

### Mask-R$^2$CNN training and ellipse fitting

Mask-R$^2$CNN is trained using the gradient descent with momentum as optimizer and unitary batch size for memory constraint. We use a multi-task loss (*L*), computed on each ROI, that is defined as $L = \alpha L_{\mathrm{cls}} + \beta L_{\mathrm{box}} + \gamma L_{\mathrm{df}}$ where $L_{\mathrm{cls}}$, $L_{\mathrm{box}}$ and $L_{\mathrm{df}}$ are the classification, bounding-box and distance-field regression loss, respectively and $\alpha , \beta , \gamma$ are constants. The $L_{\mathrm{cls}}$ and $L_{\mathrm{box}}$ are identical to those defined in Mask-RCNN. The $L_{\mathrm{df}}$ is the root mean square error computed between the distance-field ground truth and prediction, as in our previous work [4]. We trained Mask-R$^2$CNN starting from the backbone pretrained on the COCO dataset, using weights publicly available online.^2^ We then performed transfer learning by training the Mask-R$^2$CNN heads alone, freezing the backbone. Following consideration in the literature [13], since the $L_{\mathrm{cls}}$ and $L_{\mathrm{box}}$ drop faster than $L_{\mathrm{df}}$, we further trained the distance-field regression head alone, freezing the other weights of Mask-R$^2$CNN. Finally, we trained the full Mask-R$^2$CNN. We selected the best model among epochs as the one with the lowest *L* on the validation set. Following similar approaches in the literature (e.g., [6, 14]) the output of Mask-R$^2$CNN was thresholded prior performing ellipse fitting using a geometric distance based method (i.e., ElliFit [15]), which is unconstrained, non-iterative and computationally inexpensive. From the fitted ellipse, we derived the semi-major axis length (*a*), semi-minor axis length (*b*), angle of orientation ($\theta$), and center ($x_c$, $y_c$)), as required by the *HC18 Grand Challenge* organizers.

**Table 1:** Mask-R$^2$CNN distance-field regression heads

Operator	Kernel dimension	No. filters	Output dimension
Conv	3	256	$d \times d \times 256$
Conv	3	256	$d \times d \times 256$
Conv	3	256	$d \times d \times 2566$
Conv	3	256	$d \times d \times 256$
UpSamp	$2\times 2$	–	$2d \times 2d \times 256$
Conv	2	256	$2d \times 2d \times 256$
Conv	3	256	$2d \times 2d\times 256$
Conv	3	256	$2d \times 2d \times 256$
UpSamp	2	–	$4d \times 4d \times 256$
Conv	2	256	$4d\times 4d \times 256$
Conv	3	256	$4d \times 4d \times 256$
Conv	3	256	$4d \times 4d \times 256$
UpSamp	2	–	$8d \times 8d \times 256$
Conv	2	256	$8d \times 8d \times 256$
Conv	3	256	$8d \times 8d \times 256$
Conv	3	256	$8d \times 8d \times 256$
Conv	1	1	$8d \times 8d \times 1$

*Conv* convolution, *UpSamp* upsampling, $d\times d$ *in the Output dimension column* spatial size of the squared feature map in output from the ROI align layer. The number of channels is reported, too

## Experimental setup

Mask-R$^2$CNN was developed using the data released for the *HC18 Grand Challenge*.^3^ The dataset consists of 999 training and 335 testing images acquired from 551 women at the Department of Obstetrics of the Radbound University Medical Center, Nijmegen, Netherlands [1], using both the Voluson E8 and the Voluson 730 (General Electric, Austria). Image size is $800\times 540$ pixels, with a pixel physical size ranging from 0.052 to 0.326 mm, due to sonopraphers’ adjustments when imaging fetuses at different trimesters. For each image, a sonographer manually delineated the HC by drawing an ellipse that best fitted the skull. In this work, 300 training images were used as validation set. Challenges of the testing images included different position of the head in the image, as well as varying dimension of the fetal head among the gestational trimesters. Reverberations and shadows were also present, with resulting poor head contrast.

To train Mask-R$^2$CNN, the COCO challenge annotation format was followed. Starting from the HC annotation, we generated the bounding box that bounded the HC distance field. Following [4], the distance-field ground truth was obtained with *r* equal to 100 pixels. This allowed to fully cover the head-skull section at each HC point. Prior feeding Mask-R$^2$CNN, the images were resized to $512\times 512$ pixels, using zero padding to avoid changing the image aspect ratio. Following the original Mask-RCNN implementation, and considering the HC size in the US images, the RPN anchor scales were set to [32, 64, 128, 256, 512], with an anchor ratio of [0.5, 1, 2], where 1 means that the anchor is squared. We set the ROI Align output size ($d\times d$) to $14\times 14$ and considered a total of 150 training ROIs per image, as a trade off between accuracy and memory consumption.

Mask-R$^2$CNN training was performed using gradient descent with momentum with an initial learning rate and momentum of 0.001 and 0.9, respectively. The weights of the Mask-R$^2$CNN backbone (i.e., ResNet-101) were initialized with the pre-trained COCO weights. The $\alpha , \beta , \gamma$ values were set to 1 after preliminary investigation. As introduced in section “Mask-R$^2$CNN training and ellipse fitting”, Mask-R$^2$CNN was trained as follows: 50 epochs for the heads (freezing the other layers), 50 epochs for the distance-field head (freezing the other layers) and 50 epochs for the whole network. On-the-fly data augmentation was performed using scaling, translation, rotation and shearing transformations. Thresholding prior ellipse fitting was performed using a threshold of 0.9. The HC physical length [mm] was obtained by multiplying the HC pixels for the corresponding pixel size [mm], provided by the *HC18 Grand Challenge* organizers. All the analyses were performed using *Keras*^4^ on a NVIDIA RTX 2080TI, with a Xeon e5 CPU and 128 GB RAM. The implementation of Mask-R$^2$CNN was inspired by [16].

Following the guidelines of the *HC18 Grand Challenge*, we submitted our results to the challenge platform and computed the difference (DF) [mm], absolute difference (ADF) [mm], Hausdorff distance (HD) [mm] and Dice similarity coefficient (DSC).

**Fig. 4:**
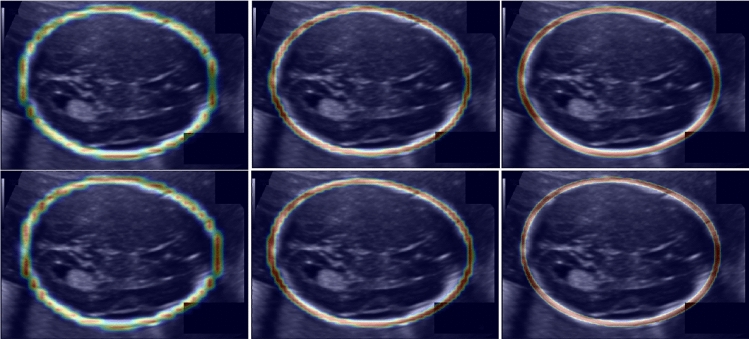
Visual samples of the predicted distance field overlapped on a test US image for each of the ablation study. A colormap is used for the predicted distance field for visualization purposes. First row (from left to right): Transp1, Transp2, Transp3. Second row (from left to right): Up-conv1, Up-conv2, Mask-R$^2$CNN

### Ablation study and comparison with the literature

The ablation study of this work is focused on the distance-field regression head. As a first study, we investigated the use of transposed convolution in the upsampling path. We considered one (Transp1, as in the original Mask RCNN work), two (Transp2) and three (Transp3) transposed convolution. We further investigated the use of upconvolution for a fair comparison with [4]: we compared the proposed upsampling path, which has 3 upconvolutions, with 1 (Up-conv1) and 2 upconvolutions (Up-conv2). This study allowed us to find the best depth of the upsampling path. It is worth noting that the network proposed in [4] has 5 up-convolutions. Nonetheless, a comparison with such a number of up-convolutions was not possible due to memory constraint. As an additional ablation study, we evaluated the performance of Mask-R$^2$CNN trained without the classification head (MaskNoClass). For fair comparison, the ablation study was performed using the same dataset split, training setting, and computational hardware.

We decided to compare the performance of Mask-R$^2$CNN against [4], which is the most similar to this work, and against [6, 8, 10, 14, 17] which follow the deep-learning paradigm and use the *HC18 Grand Challenge* dataset only. We excluded the work in [11] because it uses a portion of the training set of the *HC18 Grand Challenge* for evaluation purposes. We decided to include also the work in [1], even if it relies on handcrafted features, because it introduced the *HC18 Grand Challenge* dataset.

**Table 2:** Ablation-study results

	Absolute difference	Difference	Dice similarity coefficient	Hausdorff difference
Transp1	$2.71 \,(\pm 2.42)$	$-2.11 \,(\pm 2.95)$	$97.44 \,(\pm 1.10)$	$1.46 \,(\pm 0.83)$
Up-conv1	$2.38 \,(\pm 2.12)$	$-1.37 \,(\pm 2.88)$	$97.33 \,(\pm 1.28)$	$1.56 \,(\pm 0.82)$
Transp2	$2.69 \,(\pm 2.20)$	$-2.12 \,(\pm 2.76)$	$97.54 \,(\pm 1.10)$	$1.56 \,(\pm 0.87)$
Up-conv2	$2.05 \,(\pm 1.86)$		$97.56 \,(\pm 1.23)$	$1.48 \,(\pm 0.84)$
Transp3	$2.08 \,(\pm 2.05)$	$-0.80 \,(\pm 2.81)$	$97.83 \,(\pm 1.07)$	**1.32** (± **0.77**)
**Mask-R**$^2$**CNN**	**1.95** (± **1.92**)	$-0.31 \,(\pm 2.73)$	**97.90** (± **1.11**)	$1.45 \,(\pm 0.24)$

The best performance is highlighted in bold. The mean value, with standard deviation in brackets, is reported for each metric. All metrics but the Dice similarity coefficient are reported in mm

**Fig. 5:**
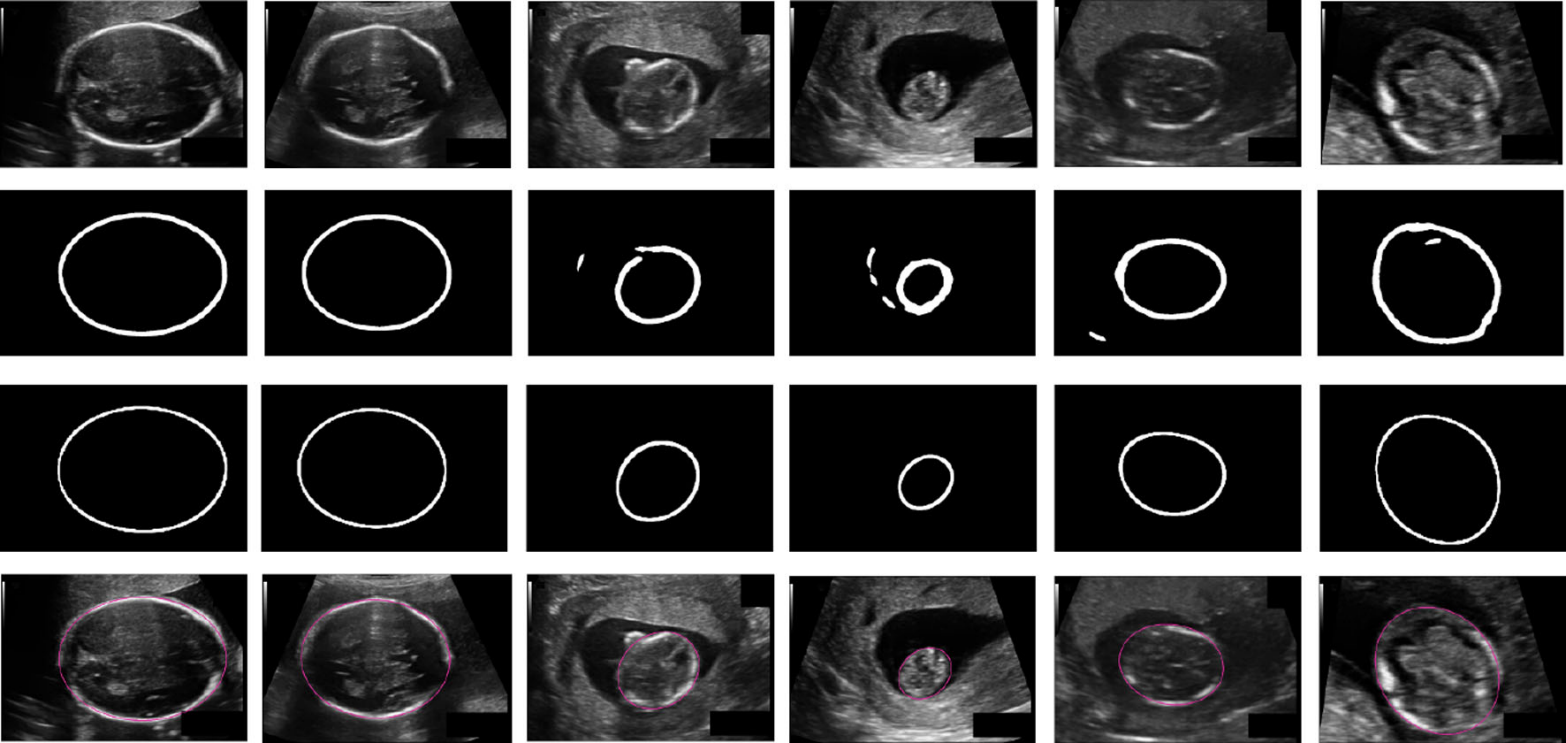
Visual samples of distance-field prediction. First row: ultrasound images, second row: prediction by [4], third row: prediction by MaskR$^2$CNN. MaskR$^2$CNN does not produce spurious predictions in challenging test images, avoiding the need of post-processing. The last row shows visual samples of fetal-head delineation with Mask-R$^2$CNN

## Results

With the ablation study, it emerged that the best performance was achieved by Mask-R$^2$CNN, with a mean AD, which is used for the final ranking of the *HC18 Challenge*, of 1.95 mm (standard deviation $= \pm 1.92$) and a mean DSC of $97.90\,(\pm 1.11)$. More specifically, we achieved a mean AD of 1.48 mm ($\pm 1.39$), 1.73 mm ($\pm 1.62$) and 3.62 mm ($\pm 2.80$) for images of first, second and third trimester, respectively. The testing time for one image was $\sim 0.9$ s. The last row of Fig. 5 shows visual samples of the HC delineation obtained by Mask-R$^2$CNN when processing challenging images in the *HC18 Challenge* test set. HC delineation is shown for fetal heads of varying size and position in the image. The Transp3 [2.08 mm ($\pm 2.05$)] and Up-conv2 [2.05 mm ($\pm 1.86$)] achieved closer AD than Mask-R$^2$CNN. The worst performance was achieved by Transp1 [2.71 mm ($\pm 2.42$)] and Transp2 [2.69 mm ($\pm 2.20$)]. Visual samples of the distance-field prediction for the ablation study are shown in Fig. 4. The distance fields obtained with Transp3 and Mask-R$^2$CNN granted the highest resolution. MaskNoClass achieved a mean DSC and AD of 82.31 and 4.14, respectively, achieving the lowest performance in the ablation study (Table 2).

For the sake of brevity, the performance relevant to the comparison with the literature are reported in terms of AD. Mask-R$^2$CNN outperformed [1] [mean AD $= 2.80$ mm (standard deviation $= \pm 3.30$)], [6] [2.12 mm ($\pm 1.87$)], [17] [2.22 mm (not available)], [14] [2.45 mm ($\pm 2.55$)], and [8] [2.33 mm ($\pm 2.21$)]. Our Mask-R$^2$CNN performed slightly worse than [10], which is the best-performing method published so far, with a difference in AD estimation of 0.14 mm. Mask-R$^2$CNN also outperformed our previous work [4] when excluding its post processing [2.33 ($\pm 3.36$)]. Nonetheless, also when including the post processing of [4], Mask-R$^2$CNN had higher DSC [97.90 ($\pm 1.11$) vs 97.76 ($\pm 1.32$)] and close AD (1.95 mm ($\pm 1.92$) vs 1.90 mm ($\pm 1.77$)]. Visual results of the distance-field regression output for Mask-R$^2$CNN and [4] are shown in Fig. 5. The method proposed in [4] produced spurious predictions both inside and outside the HC area requiring the post processing step, while Mask-R$^2$CNN produced accurate distance-field estimation.

## Discussion

In this work, an end-to-end deep-learning approach to accurately delineate HC in fetal ultrasound images was presented. The approach is based on distance field, moving our previous approach [4] forward: a modified MaskR-CNN, called Mask-R$^2$CNN, was built by replacing the segmentation head with a new head for distance-field regression. To tackle challenges related to low resolution of the predicted distance field, which would hamper accurate HC delineation, the original upsampling of Mask RCNN was replaced taking inspiration from the upsampling path of UNet. This end-to-end approach paves the way for embedding Mask-R$^2$CNN directly in US devices with short inference time, not requiring any pre- or post-processing.

Mask-R$^2$CNN was developed using the *HC18 Grand Challenge* dataset. The dataset presented multiple challenges, including poorly visible HC, different HC location and dimension as well as presence of reverberations, speckles and shadows. Despite such challenges, the results of Mask-R$^2$CNN were encouraging with a mean AD of 1.95 mm (standard deviation $\pm 1.92$), proving it to be a perfect competitor compared to other HC delineation framework in the literature. Mask-R$^2$CNN performed slightly worse on third trimester images [mean AD of 3.62 mm ($\pm 2.80$ mm) as opposed to mean AD of 1.48 mm ($\pm 1.39$ mm) obtained in the first trimester]. This may be due to the fact that image pixel dimension is higher on third-trimester images compared to those belonging to other trimesters. This was also found in [4].

With our ablation study, we showed the higher the number of up-convolutions or transposed convolutions, the more accurate the Gaussian profile regression, as shown in Fig. 4. The worst results in terms of AD were in fact achieved with Transp1 (i.e., using a single transposed convolution as in the original Mask RCNN) with a mean AD of 2.71 mm ($\pm 2.42$ mm), which was followed by Transp2 and Up-conv1 with a mean AD of 2.69 mm ($\pm 2.20$ mm) and 2.38 mm ($\pm 2.12$ mm), respectively. Up-convolutions seemed to performed slightly better compared to transposed convolutions. Hence, from our experiments up-convolution guaranteed a better distance-field output resolution than transposed convolution, allowing to have a more accurate delineation of the fetal head contours. This may not be fully appreciated considering the DSC, as it was computed from the full-head segmentation masks. Considering the AD, which is the elected metric for assessing the best method by the *HC18 Grand Challenge* organizers, it was always lower for up-convolution than for transposed convolution. This can be also appreciated from a qualitative point of view by the visual samples shown in Fig. 4. MaskNoClass achieved the lowest results in the ablation study. This may be probably explained considering that the classification loss of our Mask-R$^2$CNN had a regularization effect during training. This is in line with current considerations in the literature about multi-task learning [13, 18].

Mask-R$^2$CNN was one of the best HC delineator among state-of-the-art methods tested on the same dataset. The lower performance of [1, 14] may be explained considering that deep learning is more robust to US challenges than handcrafted-based and model-based strategies. As regard lower performance of [6], directly regressing the HC parameters, without going through an intermediate step, could be challenging for the architecture. Our approach also outperformed [8, 17], in which the HC delineation was performed as a segmentation problem. Regressing a distance-field by Mask-RCNN may be therefore a satisfactory way for HC length computation. Our approach performed slightly worse compared to [13], with a difference of 0.14 mm in AD estimation which may be probably attributed to the use of attention mechanism. However, a difference of 0.14 mm can be considered negligible compared to the whole size of the head (mean HC $=174.38$ mm among the images of the training set). Mask-R$^2$CNN also outperformed our previous work [4] when its post processing was not considered. The presence of a RPN allowed to obtain accurate predictions [especially in those images in which the uterus is particularly evident (see columns 3–6 in Fig. 5)], avoiding to rely on a post processing method to discard outliers. Nonetheless, even when the post processing was considered, the proposed framework reached a close AD, with a difference of 0.05 mm. Moreover, since the up-sampling path of [4] included one more up convolution compared to our Mask-R$^2$CNN, adding one more up-convolution is supposed to further improve the distance-field prediction. This was not tested in this work due to memory constraints.

A limitation of the proposed work can be seen in the limited size of the dataset, which however is the current benchmark in the field. A possible solution to overcame this straightforward limitation could be to exploit synthetic augmentation techniques as proposed in [19]. To directly delineate the HC, hence avoiding to rely on ellipse fitting, semantic edge localisation could be also investigated.

## Conclusions

In this work, we showed that Mask-R$^2$CNN is able to tackle the challenges of HC delineation in US images, achieving an AD of 1.95 mm, without any manual intervention nor pre or post-processing. We moved forward from our previous work [4] presenting an end-to-end architecture that can be easily embedded in US machines and used also in other clinical fields. We hope Mask-R$^2$CNN could be translated in the clinical practice to offer true support to clinicians for HC measurement.

## Footnotes

1 https://hc18.grand-challenge.org/.

2 https://github.com/matterport/Mask_RCNN/releases/download/v2.0/mask_rcnn_coco.h5.

3 https://hc18.grand-challenge.org/.

4 https://keras.io/.
